# Promoting E2F1-mediated apoptosis in oestrogen receptor-α-negative breast cancer cells

**DOI:** 10.1186/1471-2407-14-539

**Published:** 2014-07-26

**Authors:** María F Montenegro, María del Mar Collado-González, María Piedad Fernández-Pérez, Manel B Hammouda, Lana Tolordava, Mariam Gamkrelidze, José Neptuno Rodríguez-López

**Affiliations:** Department of Biochemistry and Molecular Biology A, School of Biology, Regional Campus of International Excellence “Campus Mare Nostrum”, University of Murcia, 30100 Espinardo, Murcia Spain; Durmishidze Institute of Biochemistry and Biotechnology of Agrarian University of Georgia, 0131 Tbilisi, Georgia

**Keywords:** Breast cancer, Oestrogen receptor α, E2F1, 4-Hydroxy-tamoxifen, Apoptosis

## Abstract

**Background:**

Because oestrogen receptor α (ERα) regulates E2F1 expression to mediate tamoxifen resistance in ERα-positive breast cancer cells, we aimed to define the possible roles of ERα and E2F1 in promoting the resistance of ERα-negative breast cancer cells to 4-hydroxy-tamoxifen (4OHT).

**Methods:**

This study utilised conventional techniques to demonstrate the effects of 4OHT on the expression of ERα and E2F1 and also examined the individual and combined effects of 4OHT with dipyridamole (DIPY) and 3-*O*-(3,4,5-trimethoxybenzoyl)-(-)-catechin (TMCG) on the oestrogen-negative MDA-MB-231 breast cancer cell line using viability assays, Hoechst staining, MALDI-TOF mass spectroscopy, and confocal microscopy.

**Results:**

Despite the ERα-negative status of the MDA-MB-231 cells, we observed that 4OHT efficiently up-regulated ERα in these cells and that this upregulation promoted E2F1-mediated cell growth. Because E2F1 plays a dual role in cell growth/apoptosis, we designed a therapy incorporating TMCG/DIPY to take advantage of the elevated E2F1 expression in these 4OHT-treated cells. 4OHT enhances the toxicity of TMCG/DIPY in these ERα-negative breast cancer cells.

**Conclusions:**

Because TMCG/DIPY treatment modulates the methylation status/stability of E2F1, the results demonstrate that therapies targeting the epigenetic machinery of cancer cells in the presence of overexpressed E2F1 may result in efficient E2F1-mediated cell death.

## Study design

Define the role of ERα and E2F1 in the resistance of ERα-negative breast cancer cells to 4-hydroxy-tamoxifen and develop new therapies to promote E2F1-mediated apoptosis in this type of breast cancer cell.

## Background

Human breast cancer is a heterogeneous disease with respect to molecular alterations, incidence, survival, and response to therapy. Tamoxifen has been used for the systemic treatment of patients with breast cancer for nearly four decades, and the success of this treatment is primarily dependent on the presence of oestrogen receptor-α (ERα) in the breast carcinoma. Approximately half of patients with advanced ERα-positive disease immediately fail to respond to tamoxifen, and the disease ultimately progresses to a resistant phenotype in the responding patients [1]. The possible causes for intrinsic and acquired resistance have been attributed to the pharmacology of tamoxifen, alterations in the structure and function of the ERα, interactions with the tumour environment, and genetic alterations within the tumour cells [2]. Therefore, understanding the role of ERα in the development and progression of hormone-unresponsive and receptor-dependent breast cancer is an important step in the development of future therapeutics [3].

Tamoxifen and newer selective ERα modifiers compete with estradiol to bind the ERα in multiprotein complexes that involve several co-repressor proteins. In contrast to estradiol-bound ERα, the tamoxifen-ERα complex is typically unable to promote tumour growth due to altered gene transcription and nongenomic activities of the ERα [2]. *In vitro* studies have shown that anti-oestrogen treatment of breast cancer cells can induce growth arrest via the induction of the cyclin-dependent kinase inhibitors p21 and p27 [4, 5] and cell death by mechanisms that are still being defined [6, 7]. The growth inhibitory effects of anti-oestrogens in ERα-positive breast cancer cells are profound, and this allowed early demonstration of a G1 phase site of action for anti-oestrogens [8, 9]. Studies using synchronized cells demonstrated that cells were more sensitive to oestrogens and anti-oestrogens in the early G1 phase, immediately following mitosis [10], compatible with a model whereby oestrogens and anti-oestrogens acting via the ERα regulate the rate of progression through the early G1 phase of the cell cycle. Many studies have been published characterising the multiple mechanisms of anti-oestrogen resistance, and extensive reviews of this topic are available [1, 2, 11, 12]. These studies underscore the involvement of numerous signalling pathways in ERα-regulated breast cancer cell growth and suggest novel targets to improve the efficacy of anti-oestrogen therapy. However, because tamoxifen and its derived metabolite 4-hydroxy-tamoxifen (4OHT) are specifically active against ERα-positive breast cancer cells, the effects of these drugs in ERα-negative cells are not well understood. However, it has recently been indicated that 4OHT promoted the proliferation of ERα-negative breast cancer cells via the stimulation of MAPK/ERK and Cyclin D1 expression [13].

In a recent study, we observed that a combined therapy designed to uncouple adenosine metabolism using dipyridamole (DIPY) in the presence of a new synthetic antifolate [3-*O*-(3,4,5-trimethoxybenzoyl)-(-)-catechin; TMCG] simultaneously and efficiently blocked both the folic and methionine cycles in breast cancer cells, resulting in massive cell death [14]. The TMCG/DIPY combination acted as an epigenetic treatment that reactivated RASSF1A expression and induced E2F1-mediated apoptosis in breast cancer cells. In addition to modulating DNA methylation and chromatin remodelling, this combination also induced the demethylation of the E2F1 transcription factor. Therefore, we demonstrated that the simultaneous targeting of DNA and E2F1 methylation was an effective epigenetic treatment to induce apoptosis in breast cancer cells. Importantly, the apoptotic effect of this combination was shown to be independent of the mutational status of the *p53* gene and the levels of expression of ERα, two factors that determine the sensitivity or resistance of breast cancer cells to apoptosis [15, 16].

Recently, it has been suggested that ERα regulates E2F1 expression to mediate tamoxifen resistance in ERα-positive breast cancer cells [17]. Because TMCG/DIPY treatment positively influenced E2F1-mediated cell death, we hypothesised that this combination may represent an attractive strategy to target overexpressed E2F1 in these tamoxifen-resistant cells. Consistent with this hypothesis, we observed that TMCG/DIPY treatment was highly effective against MCF7 tamoxifen-resistant cells, suggesting that this combinational therapy could be successfully used for the treatment of patients with anti-oestrogen resistant ERα-positive breast cancers. To extend the possible application of this therapy to ERα-negative breast cancers, we sought to define the roles of ERα and E2F1 in the resistance of ERα-negative breast cancer cells to 4OHT. We observed that 4OHT efficiently up-regulated ERα in MDA-MB-231 cells despite their ERα-negative status and that the upregulation of ERα promoted E2F1-mediated cell growth. Because E2F1 plays a dual role in cell growth/apoptosis, we designed a therapy incorporating TMCG/DIPY to take advantage of the elevated E2F1 expression in these 4OHT-treated cells. We observed that by modulating the posttranslational state of E2F1, the TMCG/DIPY combination was more active in the presence of 4OHT in an ERα-negative breast cancer model.

## Methods

### Reagents and antibodies

TMCG was synthesised from catechin by reaction with 3,4,5-trimethoxybenzoyl chloride [18]. DIPY, 4OHT, U0125, and fulvestrant were obtained from Sigma-Aldrich (Madrid, Spain). Antibodies against the following proteins were used: β-Actin (Sigma; Monoclonal clone AC-15), phospho-ATM (Ser^1981^) (Millipore, Madrid, Spain; Monoclonal clone 10H11.E12), phospho-Chk2 (Thr^68^) (Millipore; Monoclonal clone E126), E2F1 (Millipore; Monoclonal clones KH20 and KH95), ERα (Millipore; Monoclonal clone F3-A), and phospho-H2AX (Ser^139^) (Millipore; Monoclonal clone JBW301).

### Cell culture and apoptosis assays

The MCF-7 and MDA-MB-231 human breast cancer cell lines were purchased from the American Type Culture Collection (ATCC) and were routinely authenticated with genotype profiling according to ATCC guidelines. The cells were maintained in the appropriate culture medium supplemented with 10% foetal calf serum and antibiotics. For experiments in hormone-deprived conditions cells were maintained for three days in phenol red-free DMEM plus 2.5% dextran-charcoal-stripped foetal calf serum (Life Technologies, Barcelona, Spain) and then they were treated in the presence or absence of 4OHT. Cell viability was evaluated by a colourimetric assay for mitochondrial function using the 2,3-Bis(2-methoxy-4-nitro-5-sulfophenyl)-2H-tetrazolium-5-carboxanilide (XTT; Sigma) cell proliferation assay. For this assay, cells were plated in a 96-well plate at a density of 1,000-2,000 cells/well. The compounds were added once at the beginning of each experiment. The Hoechst staining method was used to detect apoptosis. Replicate cultures of 1 × 10^5^ cells per well were plated in 6-well plates. The cells were subjected to the indicated treatments for 72 h. After changing to fresh medium, the cells were incubated with 5 μL of Hoechst 33342 solution (Sigma) per well at 37°C for 10 min and then observed under a fluorescence microscope. Strong fluorescence was observed in the nuclei of apoptotic cells, while weak fluorescence was observed in the non-apoptotic cells. The quantification of apoptotic cells was performed by counting the cells in four random fields in each well.

### PCR analysis

mRNA extraction, cDNA synthesis, and conventional and semiquantitative real-time PCR (qRT-PCR) were performed as previously described [19]. The primers were designed using Primer Express version 2.0 software (Applied Biosystems, Foster City, CA, USA) and synthesised by Life Technologies. The following primers for human genes were used: *β-Actin* (forward: 5′-AGA AAA TCT GGC ACC ACA CC-3′; reverse: 5′-GGG GTG TTG AAG GTC TCA AA-3′), *Apaf1* (forward: 5′-GCT CTC CAA ATT GAA AGG TGA AC-3′; reverse: 5′-ACT GAA ACC CAA TGC ACT CC-3′), *E2F1* (forward: 5′-GAG GTG CTG AAG GTG CAG AAG-3′; reverse: 5′-TTG GCA ATG AGC TGG ATG C-3′), *ERα* (forward: 5′- TGG GCT TAC TGA CCA ACC TG -3′; reverse: 5′- CCT GAT CAT GGA GGG TCA AA -3′), and *p73* (forward: 5′-TGG AAC CAG ACA GCA CCT ACT TCG-3′; reverse: 5′-CAG GTG GCT GAC TTG GCC GTG CTG-3′).

### ChIP assays

A chromatin immunoprecipitation (ChIP) assay was performed using the Magna ChIP™ G kit from Millipore according to the manufacturer’s instructions. Briefly, MDA-MB-231 cells were formaldehyde cross-linked, and the DNA was sheared by sonication to generate fragments with an average size of 300 to 3,000 bp. The chromatin was then incubated with anti-ERα or mouse IgG antibodies. DNA from lysates prior to immunoprecipitation was used as a positive input control. After washing, elution, and DNA purification, the DNA solution (2 μl) was used as a template for qRT-PCR amplification using specific human primers. The following primer sequences were used for ChIP-PCR: *E2F1* (forward: 5′-GCA AGT TGA GGA TGG AAG AGG TG-3′; reverse: 5′-TGG GGA CAC GGG AAC ATA GG-3′), *ERα* (forward: 5′-ACC TTA GCA GAT CCT CGT-3′; reverse: 5′-GCT GCT GGA TAG AGG CTG A-3′), and *GAPDH* (forward: 5′-CAA TTC CCC ATC TCA GTC GT-3′; reverse: 5′-TAG TAG CCG GGC CCT ACT TT-3′).

### Stealth RNA transfection

Specific Stealth siRNAs for E2F1 (HSS103015, HSS103016, and HSS103017) were obtained from Life Technologies and transfected into MDA-MB-231 cells using Lipofectamine 2000 (Life Technologies). The treatments were started 24 h after siRNA transfection. Stealth RNA negative control duplexes (Life Technologies) were used as control oligonucleotides, and the ability of the Stealth RNA oligonucleotides to knock down the expression of E2F1 was analysed by western blot 24 h after siRNA transfection.

### Western blot analysis

Whole cell lysates were collected by adding SDS sample buffer. After extensive sonication, the samples were boiled for 10 min and subjected to SDS-PAGE. The proteins were then transferred to nitrocellulose membranes and analysed by immunoblotting (ECL Plus, GE Healthcare, Barcelona, Spain).

### Immunoprecipitation and MALDI-TOF mass spectroscopy

For immunoprecipitation assays, approximately 2.5 × 10^7^ MDA-MB-231 cells were lysed in 500 μl of lysis buffer (50 mM Tris, pH 8.0, 300 mM NaCl, 0.4% NP40, and 10 mM MgCl_2_) supplemented with protease and phosphatase inhibitor cocktails (Sigma), 2.5 μM trichostatin (a potent deacetylase inhibitor), and 50 μM 2PCPA (an irreversible inhibitor of lysine-specific demethylase 1, LSD1). The cell extracts were cleared by centrifugation (20,000 × g for 15 min) and then diluted with 500 μl of dilution buffer (50 mM Tris, pH 8.0, 0.4% NP40, and 2.5 mM CaCl_2_) supplemented with protease and phosphatase inhibitor cocktails, DNase I (Sigma), 2.5 μM trichostatin and 50 μM 2PCPA. The extracts were pre-cleared by 30-min incubations with 20 μl of PureProteome Protein G Magnetic Beads (Millipore) at 4°C with rotation. The E2F1 antibody was then covalently coupled to Dynabeads® (Life Technologies) and added to the pre-cleared extracts. After immunoprecipitation and elution, the bound proteins were digested with trypsin according to standard procedures [20]. The data were recorded and processed with Agilent MassHunter Workstation Software to obtain the Peptide Mass Fingerprint (PMF). The resulting PMF mass spectra were searched against the E2F1 protein sequence with carbamidomethylation of cysteine as a fixed modification and phosphorylation of serine residues as variable modification. The peptide mass tolerance was set to 50 ppm, and a maximum of three missed cleavages was allowed.

### Microscopy

Confocal microscopy was carried out using a Leica TCS 4D confocal microscope (Wetzlar, Germany). For indirect immunofluorescence studies, preparations of the cells on glass slides were fixed with cold acetone for 5 min and then washed with PBS. The cells were incubated with 3% bovine serum albumin (BSA) for 20 min and then 2 h at room temperature with specific primary antibodies (diluted 1:200 in PBS containing 1% BSA). The cells were washed three times in PBS and incubated for 1 h at room temperature with Alexa Fluor Dyes as secondary antibodies (Life Technologies). After 3 washes with PBS, the cells were incubated with 0.01% 4′-6-diamidino-2-phenylidene (DAPI; Sigma) in water for 5 min. To ensure antibody specificity, primary antibodies were replaced with specific IgGs (diluted 1:200) in negative control reactions.

### Statistical analysis

In all experiments, the mean ± standard deviation (SD) values from three to five determinations in triplicate were calculated. Statistically significant differences were evaluated using a Student’s *t*-test. Differences were considered to be statistically significant at P < 0.05.

## Results and discussion

### 4OHT up-regulates ERα expression in ER-negative breast cancer cells

**Figure 1:**
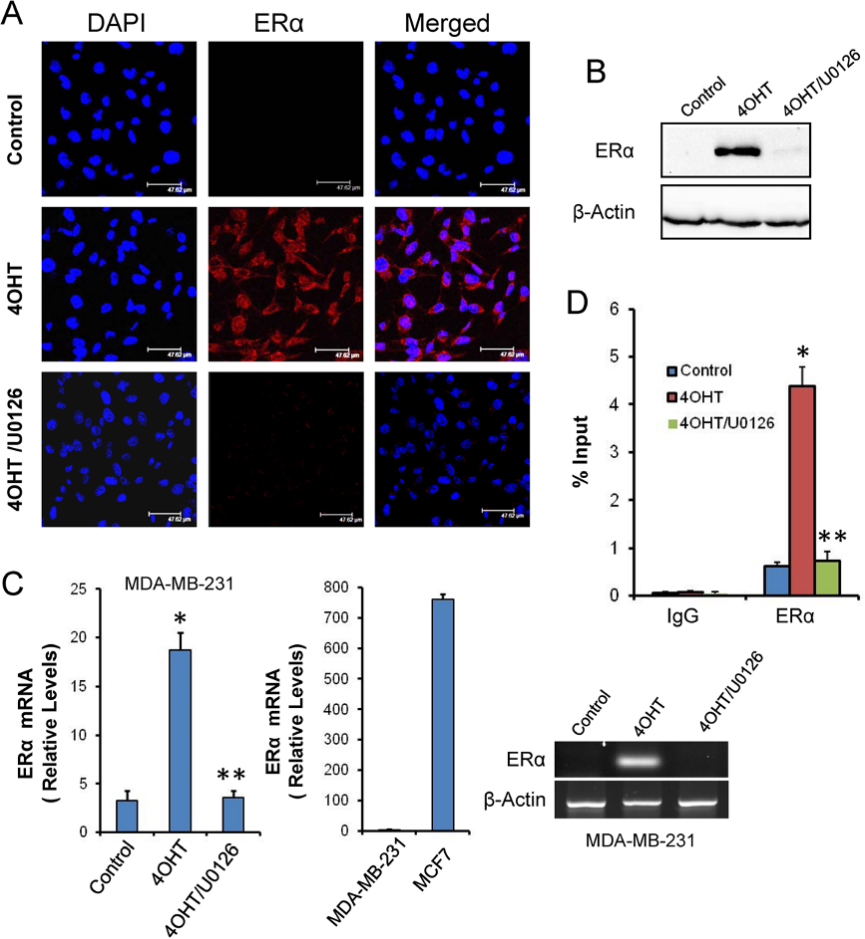
**4OHT induces the expression of ERα in MDA-MB-231 cells.** MDA-MB-231 cells were treated for 3 days with vehicle (control), 1 μM 4OHT or a combination of 1 μM 4OHT and 10 μM U0126 (4OHT/U0126). **(A)** ERα protein expression (red) in MDA-MB-231 cells was assayed by confocal microscopy. The nuclei were counterstained with DAPI (blue), and the merged images are shown. **(B)** ERα protein expression in MDA-MB-231 cells was assayed by western blot analysis. **(C)** Semiquantitative determination of ERα mRNA in breast cancer cells. The histograms were obtained by real-time PCR and represent the number of copies of mRNA for every 1 × 10^6^ copies of β-actin. **(D)** The *In vivo* binding of ERα protein to the *ERα* promoter as determined by ChIP assays. Immunoprecipitation using anti-ERα or an IgG was performed in triplicate. The results are presented as the percentage of the input DNA. In this figure: *indicates that P < 0.05 when compared with untreated controls, and **indicates that the differences were significant (P < 0.05) when compared to 4OHT-treated cells but non-significant when compared to untreated controls.

Breast cancer cells are classified as either ER-positive or ER-negative, depending on the presence or absence of ER, particularly ERα. Although there are two known isoforms of ER, ERα and ERβ [21], much of what we know about ER-dependent breast cancer has focused on ERα. In this report, confocal microscopy experiments and western blot assays (Figure 1A and B) indicated that the MDA-MB-231 breast cancer cell line did not express ERα, which was in accordance with its ERα-negative status. However, semiquantitative real-time PCR designed to detect ERα mRNA showed a consistent expression of this mRNA transcript, estimated to be approximately 3.3 copies of ERα mRNA for every 10^6^ copies of β-actin (Figure 1C). Although the specificity of the amplification of this ERα PCR product was verified by melting curve analysis, this product was barely detectable by agarose gel electrophoresis (Figure 1C). Although ERα mRNA was detected in MDA-MB-231 cells, its expression levels, as expected, were much lower than in the ERα-positive cell line, MCF7 (approximately 761 copies of ERα mRNA for every 10^6^ copies of β-actin; Figure 1C).

Because the response of ER-negative breast cancer cells to ER antagonists with respect to ERα expression is not well known, we aimed to determine whether 4OHT can modulate ERα expression in MDA-MB-231 cells by analysing the ERα mRNA levels in 4OHT-treated cells. As observed in Figure 1C, 4OHT significantly induced the expression of ERα by approximately 6-fold compared to untreated control cells. The ERα PCR product was sequenced, and the published ERα sequence was confirmed (data not shown). Importantly, this increase in ERα mRNA was accompanied by a significant increase in ERα protein as determined by confocal microscopy and western blot experiments (Figure 1A and B). There are at least two possible mechanisms by which ERα gene expression may be lost in ERα-negative breast cancer cells. First, the activators necessary for ERα transcription may not be available or the transcriptional repressors may predominate. Alternatively, the ERα gene may be selectively methylated and inaccessible to the existing transcriptional activators [22]. Because 4OHT has not been identified as a demethylating agent, it seems more probable that this drug affected the pattern of ERα transcription by modulating the range of its activators/repressors on its corresponding promoter. It is well established that the MAPK-mediated hyperphosphorylation of ERα can contribute to resistance to tamoxifen in breast cancer and that serine 118 in ERα is an important residue for the stimulation of ERα activity by the selective ER modulator 4OHT [23]. To demonstrate the involvement of the MAPK signalling pathway in the 4OHT-induced up-regulation of ERα, we used U0126, a MAP-ERK kinase (MEK) 1/2 inhibitor. As observed in Figure 1A-C, U0126 inhibited ERα mRNA and protein expression in cells co-treated with 4OHT. ChIP assays also indicated that 4OHT increased the occupancy of ERα on its own promoter and that U0126 completely abolished this occupation (Figure 1D). It is well known that phosphorylation of ERα at specific residues can stimulate ERα activity in a ligand-independent manner [23]. By this mechanism of action ERα is phosphorylated by active kinases, thereby activating ERα to dimerise, bind DNA, and regulate genes [24]. Taken together, these results indicated that 4OHT may promote this ERα ligand-independent pathway in ERα-negative breast cancer cells, activating the MAPK-mediated phosphorylation of ERα, which may contribute to its own expression (autoregulation) [25].

### The up-regulation of ERα by 4OHT contributes to anti-oestrogen resistance in ER-negative breast cancer cells

**Figure 2:**
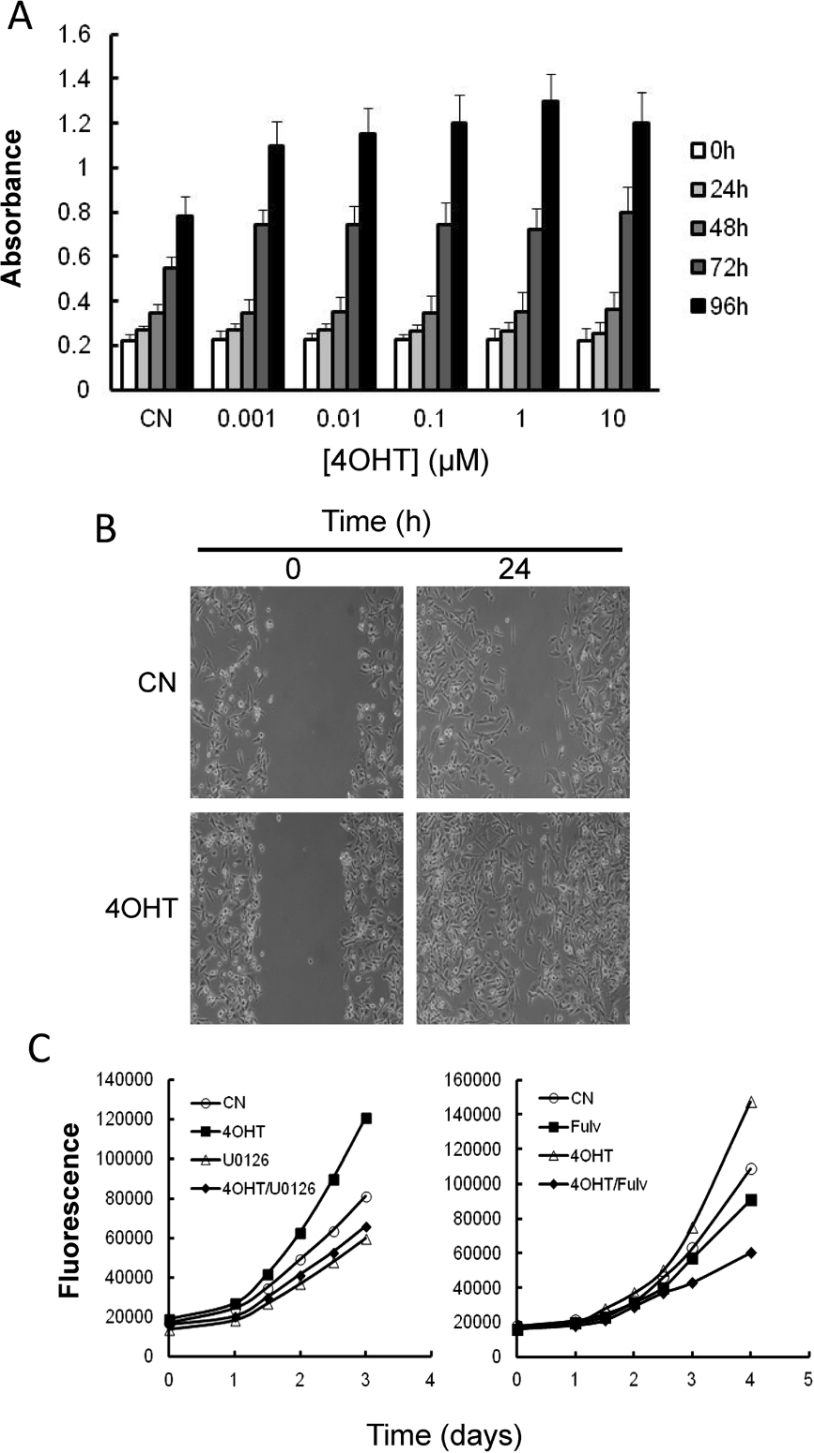
**Effects of 4OHT on the growth of MDA-MB-231 cells. (A)** Concentration- and time-dependent effects of 4OHT on the growth of MDA-MB-231 cells. The cells were plated in 96-well plates and treated with different concentrations of 4-OHT. After the indicated times, the viability of the cells was evaluated using the XTT assay. The absorbance reading at 450 nm was proportional to cell viability. The differences in cell growth after 72 h were statistically significant (P < 0.05) at all the studied 4OHT concentrations when compared to untreated controls (CN). **(B)** The migratory ability of MDA-MB-231 cells treated with vehicle (control; CN) or with 1 μM 4OHT was analysed using a wound healing assay. **(C)** The effects of U0126 (10 μM) and fulvestrant (10 μM) on the growth of MDA-MB-231 cells treated with vehicle (control; CN) or with 1 μM 4OHT.

ERα-positive breast cancers generally have a better prognosis and are often responsive to anti-oestrogen therapy, which was the first example of a successful therapy targeting a specific protein, the ERα. Unfortunately, ERα-negative breast cancers are more aggressive and unresponsive to anti-oestrogens. Although the transformation of ERα-negative into ERα-positive cells by gene therapy or ERα gene re-expression are common strategies to restore anti-oestrogen responsiveness [26], we observed in this study that MDA-MB-231 ERα-negative cells were intrinsically resistant to 4OHT despite the overexpression of ERα. Proliferation assays to determine the concentration and time-dependent effects of 4OHT on MDA-MB-231, showed that this drug stimulated cancer cell proliferation at concentrations as low as 1 nM (Figure 2A). Stimulation of cancer cell proliferation, in the presence of 4OHT, was also observed in hormone-deprived conditions, which indicated that this effect was independent of oestrogens in the culture medium. This class of resistance to tamoxifen was firstly discovered by Gottardis and Jordan [27] in MCF7 cells and the results agree with additional observation in which long term tamoxifen treatment of MDA-MB-231 cells increased their growth and their aggressiveness in animal tumours [28].

ERα-positive tumours are more differentiated and have lower metastatic potential than ERα-negative tumours [29]. Because this suggests a protective role of the oestrogen receptor in tumour progression and metastasis, we next examined whether the 4OHT-induced expression of ERα resulted in a decreased metastatic potential of MDA-MB-231. Using a wound healing assay, we observed that the treatment of MDA-MB-231 with 4OHT greatly increased the migratory ability of these cells (Figure 2B). These results indicated that the re-expression of ERα in ERα-negative breast cancer cells may promote cell growth and the resistance of these cells to anti-oestrogen therapy.

To determine whether ERα plays a role in 4OHT resistance, we examined the effect of a decreased level of ERα on the growth of MDA-MB-231 cells. Because the treatment of these cells with U0126 clearly inhibited the ERα expression induced by 4OHT (Figure 1A-C), we first analysed whether this drug also inhibited 4OHT-induced cell proliferation (Figure 2C). As shown in this Figure, U0126 reduced the growth of cells that were treated with 4OHT and untreated cells to the same extent. These results indicated that the MAPK cascade controls the proliferation of this breast cancer cell line. It is well known that membrane ER-α plays a role in the temporal coordination of phosphorylation/dephosphorylation events for the ERKs in breast cancer cells [30]. Although at the moment we unknown the mechanism by which 4OHT activates the MAPK cascade, it is tempting to speculate that in ERα-negative breast cancer cells may exist some levels of membrane-bound receptor. Related to this, our group (in this study) and others [31] have detected consistent expression of ERα-mRNA in MDA-MB-231 cells; however, whether this expression may be related to membrane-bound ERα is actually unknown. More recently, Zhang and co-workers [13] indicated that 4OHT promoted the proliferation of ERα-negative breast cancer cells via the stimulation of the MAPK/ERK pathway, which is mediated by ERα-36. Therefore, understand how 4OHT activates the MAPK cascade in ERα-negative breast cancer cells will require further studies.

As a second approximation and to reduce the endogenous levels of ERα, we treated MDA-MB-231 cells with fulvestrant. Fulvestrant is a potent anti-oestrogen that possesses extremely high ERα binding affinity and has two major effects on ERα signalling. First, it blocks ERα signalling by inhibiting receptor dimerisation and nuclear localisation and, second, it blocks ERα expression and ERα-mediated gene transcription [32]. In addition, binding of fulvestrant to the ERα has been proposed to induce proteasomal degradation of the aberrant receptor complex [33]. As observed in Figure 2C, treatment with fulvestrant reduced the growth of cells treated with 4OHT. All together, these experiments support the notion that the effects of 4OHT on cell growth promotion in MDA-MB-231 cells were dependent on an intact ERα signalling.

### ERα regulates E2F1 expression in ER-negative breast cancer cells to mediate 4OHT resistance

**Figure 3:**
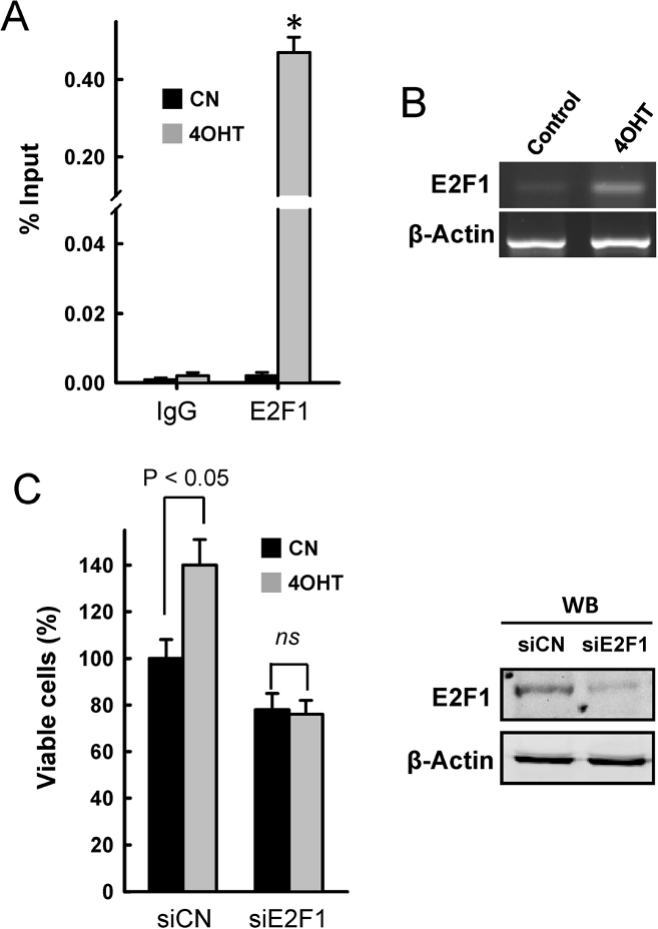
**ERα regulates E2F1 expression in MDA-MB-231 cells. (A)** The *In vivo* binding of ERα protein to the *E2F1* promoter. ChIP assays using untreated MDA-MB-231 cells (control; CN) or those treated for 3 days with 1 μM 4OHT. Immunoprecipitation using anti-ERα or IgG was performed in triplicate. The results are presented as the percentage of the input DNA. *indicates that P < 0.05 when compared to the untreated controls. **(B)** PCR was used to detect E2F1 mRNA in untreated MDA-MB-231 cells or MDA-MB-231 cells treated for 3 days with 1 μM 4OHT. The data shown here are from a representative experiment repeated four times with similar results. **(C)** MDA-MB- cells 231 transfected with siCN or siE2F1 were treated for 3 days with 1 μM 4OHT when indicated, and cell growth was monitored 4 days after transfection (3 days after 4OHT treatment). The expression of E2F1 protein was monitored by western blot (WB), and β-actin was used as a loading control. The statistical values represent data from four replicate samples.

Recently, it has been proposed that ERα regulates E2F1 expression to mediate tamoxifen resistance in ERα-positive cells [17]. The overexpression of E2F1 has been shown to be sufficient to promote breast cancer proliferation [34] by regulating the expression of cyclins and Cdks to mediate the G_1_-S transition [35]. Although the E2F1 promoter lacks the classic oestrogen-response element 5′-GGTCAnnnTGACC-3′, ERα is known to interact with several other transcription factors, including Sp1 [36]. In fact, it has been clearly demonstrated that tamoxifen increases ERα/Sp1 interactions, modulating E2F1 expression in MCF7 tamoxifen-resistant cells [17]. Therefore, we performed a ChIP assay to determine whether 4OHT can promote the recruitment of ERα to the E2F1 promoter. The results shown in Figure 3A indicated that compared to the control, 4OHT substantially increased the occupancy of ERα on the E2F1 promoter (from 0.002% in untreated cells to 0.47% in 4OHT-treated cells with respect to an input control). Consistent with the ChIP and proliferation data, semiquantitative RT-PCR analysis indicated that the expression levels of E2F1 were markedly increased after exposure to 4OHT conditions. For these experiments, control cells and those subjected to 72 h of 4OHT treatment were harvested for gene expression analysis (Figure 3B). Finally, to determine whether E2F1 contributed to the increased proliferation after 4OHT exposure, we silenced E2F1 expression in MDA-MB-231 cells using specific siRNAs (Figure 3C). Although E2F1 silencing diminished the proliferation of vehicle-treated cells, 4OHT did not increase cellular proliferation in E2F1-depleted cells. Collectively, these results suggest that, in response to 4OHT, ERα regulates E2F1 expression to mediate both cellular growth and anti-oestrogen resistance. Since the 4OHT:ERα complex usually weakly binds to any promoter, these results also indicated that 4OHT might promote the ERα ligand-independent pathway in which phosphorylated ERα may control the expression of several genes, including E2F1.

### Designing a combined therapy to enhance the E2F1 pro-apoptotic signalling in 4OHT-treated ER-negative breast cancer cells

**Figure 4:**
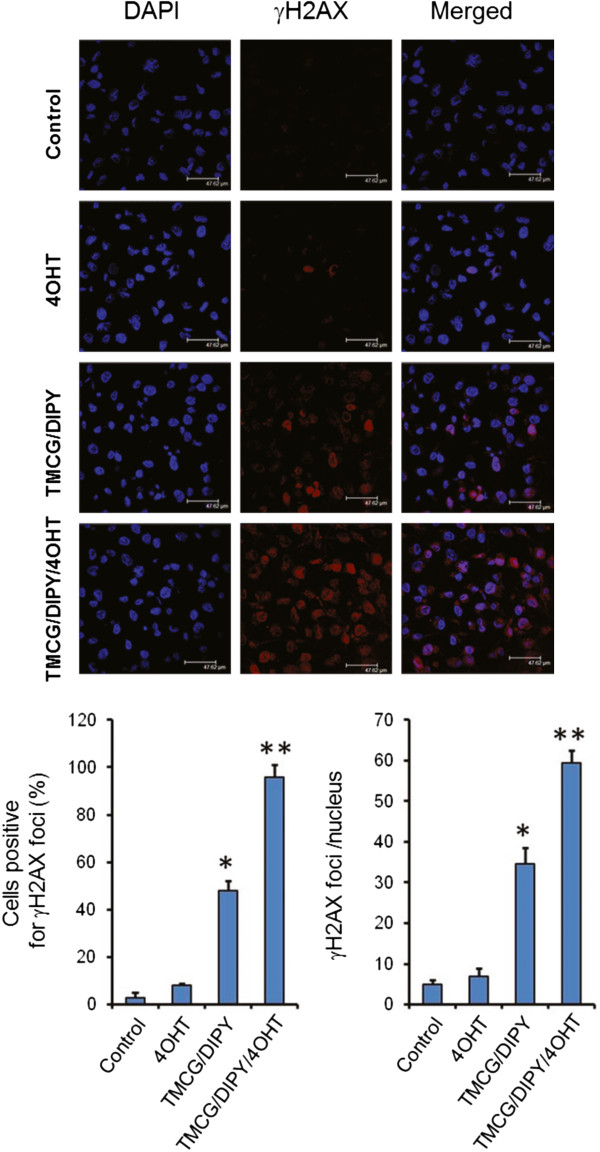
**The effects of several individual and combined treatments on γH2AX foci formation in MDA-MB-231 cells.** MDA-MB-231 cells were treated for 3 days with vehicle (control), 1 μM 4OHT, 10 μM TMCG and 5 μM DIPY (TMCG/DIPY) or with the triple combination TMCG/DIPY/4OHT at the specified concentrations. MDA-MB-231 cells were examined by immunofluorescence for γH2AX foci (red) and DAPI (blue). The histograms represent the positive γH2AX foci cells and the number of γH2AX foci per nucleus in γH2AX focus-positive cells. In both cases, *indicates that P < 0.05 when compared to untreated cells subjected to 4OHT treatment and **indicates that P < 0.05 when compared to untreated cells subjected to 4OHT and TMCG/DIPY treatment.

**Figure 5:**
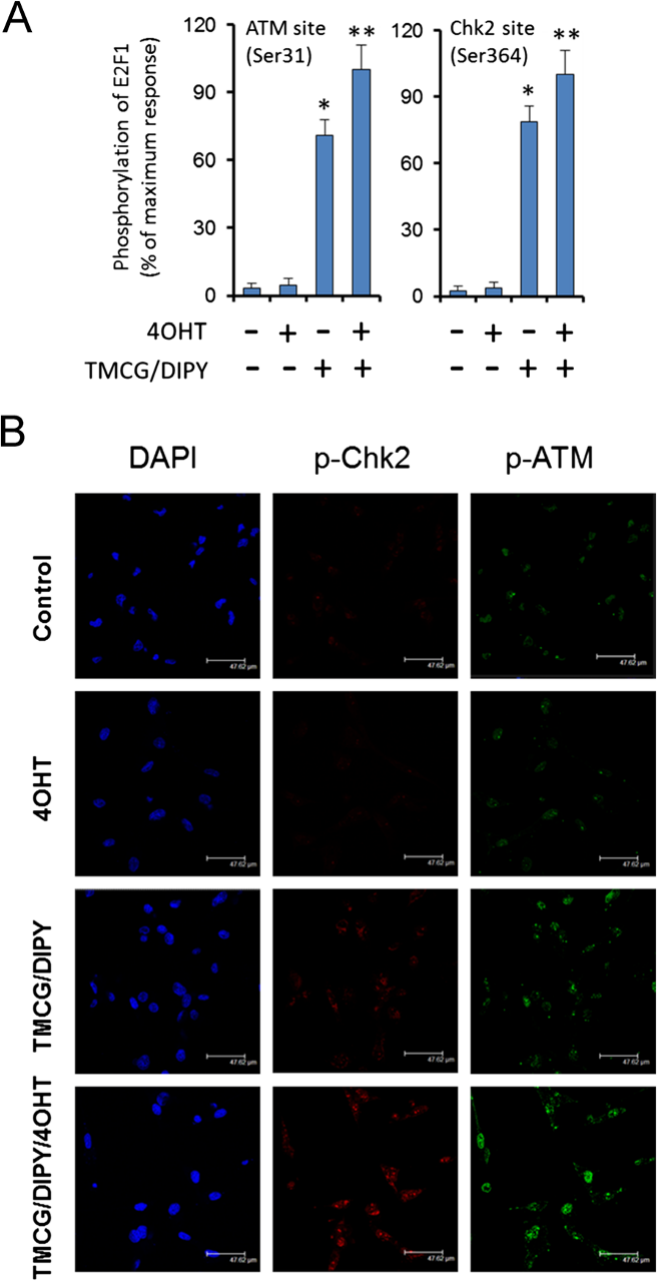
**The effects of several individual and combined treatments on E2F1 phosphorylation and ATM/Chk2 activation in MDA-MB-231 cells. (A)** The phosphorylation status of E2F1 in response to treatments was determined by MALDI-TOF mass spectroscopy. Peptides were analysed in untreated MDA-MB-231 cells (control) or those treated for 3 days with 1 μM 4OHT; 10 μM TMCG and 5 μM DIPY (TMCG/DIPY); or 10 μM TMCG, 5 μM DIPY, and 1 μM 4OHT (TMCG/DIPY/4OHT). The relative intensities of specific tryptic peptides were normalised to an internal matrix control. **(B)** MDA-MB-231 cells subjected to the above-described treatment were analysed by immunofluorescence for p-ATM (green), p-Chk2 (red) and DAPI (blue).

The E2F family of transcription factors plays a key role in the regulation of cell growth, apoptosis, and oncogenic transformation by mediating the timely expression of genes involved in these processes [37]. Although the overexpression of E2F1 has been directly correlated with its pro-apoptotic activity [38], more recent data indicated that the control of E2F1-dependent cell growth or pro-apoptotic pathways is more related to the posttranslational processing of this transcription factor [39, 40]. Recently, negative crosstalk between methylation and other post-translational modifications of E2F1, such as acetylation and phosphorylation, has been described [39, 40]. Thus, methylated E2F1 is prone to ubiquitination and degradation, whereas the demethylation of E2F1 favours its P/CAF-dependent acetylation at lysine residues 117, 120, and 125 [41]. Whether acetylated E2F1 binds to the promoter of genes required for S phase (to allow cell growth) or to the promoters of proapoptotic genes (to induce cell death) may depend on its subsequent phosphorylation by specific kinases. Thus, in response to severe DNA damage, the hyperacetylated E2F1 protein is stabilised through direct phosphorylation by Chk2 at Ser^364^ or ATM kinase at Ser^31^ [42, 43]. Although 4OHT induced the ERα-mediated expression of E2F1 (Figure 3B), the treatment of MDA-MB-231 cells with this drug did not result in visible DNA double strand breaks (DSBs) (as determined by the absence of phosphorylation of histone H2AX at Ser^139^, Figure 4). Therefore, consistent with these results, 4OHT did not increase the phosphorylation of E2F1 at Serines 31 and 364 (Figure 5A). Trypsin digests of E2F1 immunoprecipitated from untreated and 4OHT-treated MDA-MB-231 cells primary yielded the peptides (R)LLDSSQIVIISAAQDASAPPAPTGPAAPAAGPC(Carbamidomethyl)DPDLLLFATPQAPRPTPSAPRPALGRPPVK(R) (measured m/z = 6275.2592) and (R)MGSLRAPVDEDR(L) (measured m/z = 1346.5131), which correspond to the non-phosphorylated Ser^31^- and Ser^364^-containing peptides, respectively. Taken together, these data strongly agree with the results showing the resistance of MDA-MB-231 cells to 4OHT, which resulted in an enhancement of cell growth but not visible apoptosis.

Recently, we observed that the TMCG/DIPY combination acted as an epigenetic treatment that reactivated RASSF1A expression and induced apoptosis in breast cancer cells. In addition to modulating DNA methylation and chromatin remodelling, this combination also induced the demethylation of the E2F1 transcription factor [14]. Because these agents modulated multiple aspects of breast cancer cell metabolism and survival, including the folic acid and methionine cycles and the methylation status of cells, we observed that the TMCG/DIPY combination induced the formation of DNA DSBs characterised by phosphorylation of histone H2AX at Ser^139^ (γH2AX) and its accumulation in nuclear foci (Figure 4). Here, we observed that this therapy also induced the substantial activation of ATM and Chk2, which resulted in nuclear focus localisation of p-ATM and p-Chk2 (Figure 5B). Consistent with these results, treatment of MDA-MB-231 cells with TMCG/DIPY resulted in an increase in E2F1 phosphorylation at Ser^31^- and Ser^364^ (Figure 5A); [14]. Because TMCG/DIPY treatment positively influenced E2F1-mediated cell death [14], we hypothesised that this combination might represent an attractive strategy to target overexpressed E2F1 in 4OHT-treated ER-negative breast cancer cells.

**Figure 6:**
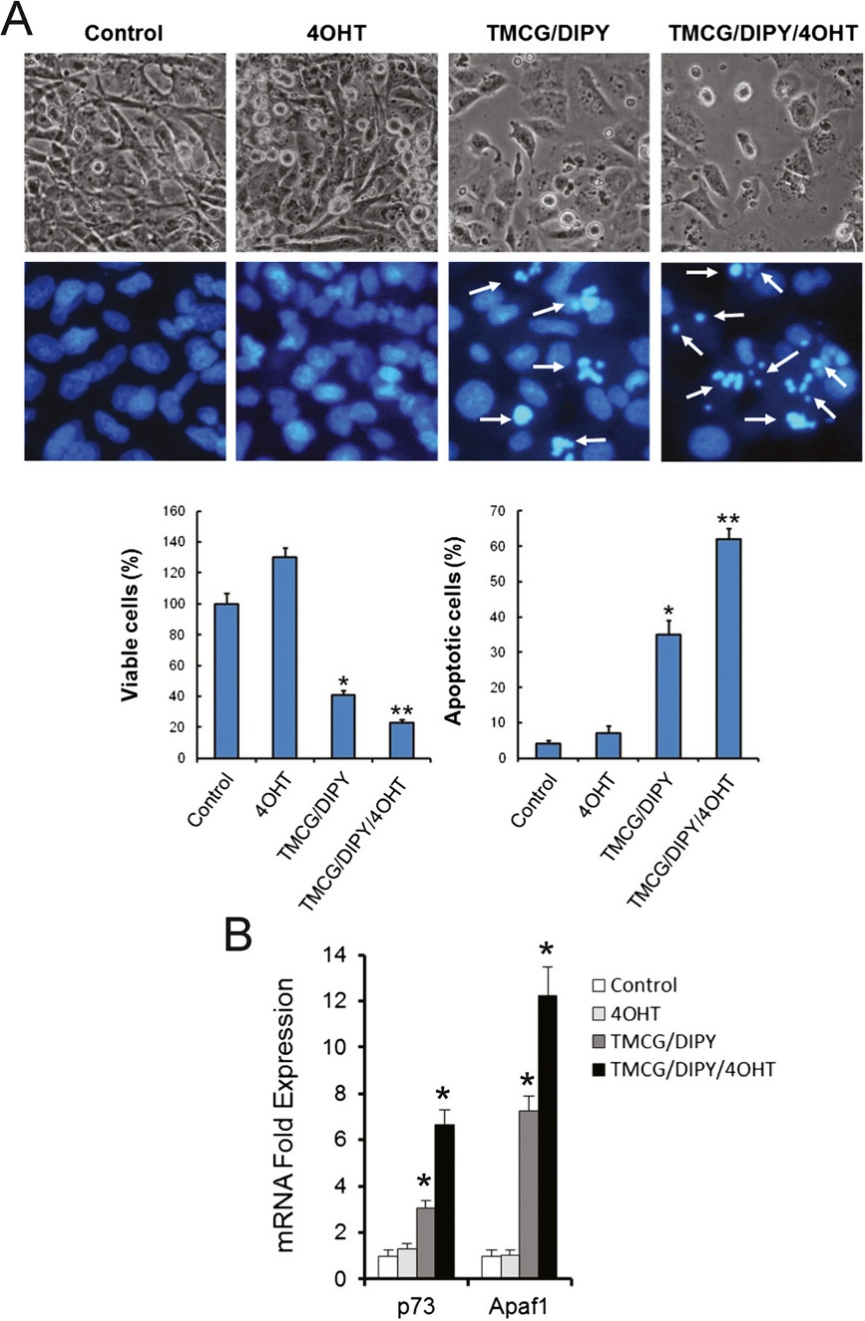
**4OHT enhances TMCG/DIPY toxicity in MDA-MB-231 cells.** In all cases, the cells were treated for 3 days with vehicle (control); 1 μM 4OHT; 10 μM TMCG and 5 μM DIPY (TMCG/DIPY); or 10 μM TMCG, 5 μM DIPY, and 1 μM 4OHT (TMCG/DIPY/4OHT). **(A)** The images show the effect of the 4OHT, TMCG/DIPY and TMCG/DIPY/4OHT treatments on the morphology (upper panels) and apoptosis (lower panels) of MDA-MB-231 cells as determined by bright-field microscopy and fluorescence microscopy after DNA staining with Hoechst 33342. The histograms represent the percentage of viable and apoptotic cells calculated from the images shown above. *P < 0.05 compared to the untreated controls; **P < 0.05 compared to TMCG/DIPY-treated cells. **(B)** Semiquantitative determination of p73 and Apaf1 mRNAs in MDA-MB-231 after different treatments. The estimated relative levels of the specific mRNA with respect to that of β-actin mRNA were calculated and then compared with respect to the expression levels in untreated controls. *P < 0.05 compared to the untreated controls.

To test our hypothesis, MDA-MB-231 cells were treated with the triple TMCG/DIPY/4OHT combination, and the effects of this combination on cell growth and apoptosis were compared to those of the double TMCG/DIPY combination treatment. As observed in Figure 6A, addition of 4OHT to the double TMCG/DIPY combination significantly increased the number of apoptotic cells (from 35% in TMCG/DIPT-treated cells to more than 60% in cell cultures subject to the triple combination). This increase in apoptosis was also correlated with cellular damage, as indicated by an increase in the number of cells that were positive for phosphorylated histone H2AX (γH2AX) and with an augmented ratio of γH2AX per nucleus (Figure 4). The analysis of E2F1 phosphorylation at Serines 31 and 364 indicated that overexpressed E2F1 was fully phosphorylated in cells subjected to TMCG/DIPY/4OHT therapy (Figure 5A), which is consistent with the pattern of Chk2 and ATM activation (phosphorylation) in these cells (Figure 5B). Consistent with our hypothesis of TMCG/DIPY/4OHT combination inducing E2F1-mediated apoptosis, we observed a significant increase of p73 and Apaf1 mRNAs after treatment (Figure 6B).

## Conclusions

As described in the introduction, the mutational status of the *p53* gene and/or the levels of expression of ERα determine the sensitivity or resistance of breast cancer cells to apoptosis [15, 16]. The p53 tumour suppressor protein is an essential component of the cell response induced by genotoxic stresses, but the *p53* gene is inactivated or mutated in the majority of human tumours. To overcome these obstacles, genes that can compensate or bypass cell death defects regardless of the *p53* status are particularly useful. E2F1 and its proapoptotic genes represent such a group of molecules and therefore have direct implications as anti-neoplastic therapeutics for cancers lacking p53 activity. Recently, our research group has generated novel antifolate drugs that have successfully been used in combined hypomethylating therapies against melanoma [19, 44] and breast cancer [14]. Here, we present an experimental therapy that is effective in breast cancer cells independently of their p53 and ERα status, and confirm the hypothesis that the elevation of E2F1 in the presence of genotoxic stress could represent a valuable therapy against cancers [38]. Because the demethylation of E2F1 is required for its DNA damage-induced accumulation and the activation of its proapoptotic target genes (such as *p73*, *Apaf1* and *Bim*, among others), therapies to target the epigenetic machinery of cancer cells may result in utility to promote E2F1-dependent apoptosis in p53-deficient tumours. Because TMCG/DIPY treatment modulates the methylation status and stability of E2F1, we observed that the 4OHT-dependent overexpression of E2F1 positively influences E2F1-mediated cell death in ERα-negative breast cancer cells.

## Abbreviations

ATM: Ataxia telangiectasia mutated
Chk2: Checkpoint kinase 2
ChIP: Chromatin immunoprecipitation
DAPI: 4′-6-Diamidino-2-phenylidene
DIPY: Dipyridamole
ER: Oestrogen receptor
GAPDH: Glyceraldehyde 3-phosphate dehydrogenase
4-OHT: 4-Hydroxy-tamoxifen
IgG: Immunoglobulin G
XTT: 2,3-Bis(2-methoxy-4-nitro-5-sulfophenyl)-2H-tetrazolium-5-carboxanilide
TMCG: 3-*O*-(3,4,5-trimethoxybenzoyl)-(-)-catechin.
